# Mood instability is a common feature of mental health disorders and is associated with poor clinical outcomes

**DOI:** 10.1136/bmjopen-2014-007504

**Published:** 2015-05-16

**Authors:** Rashmi Patel, Theodore Lloyd, Richard Jackson, Michael Ball, Hitesh Shetty, Matthew Broadbent, John R Geddes, Robert Stewart, Philip McGuire, Matthew Taylor

**Affiliations:** 1 Department of Psychosis Studies, King's College London, Institute of Psychiatry, Psychology & Neuroscience, London, UK; 2 Department of Psychological Medicine, King's College London, Institute of Psychiatry, Psychology & Neuroscience, London, UK; 3 South London and Maudsley NHS Foundation Trust, Biomedical Research Centre Nucleus, London, UK; 4 Department of Psychiatry, University of Oxford, Oxford, UK

**Keywords:** mood instability, Clinical Record Interactive Search (CRIS), natural language processing (NLP), text mining, electronic health record (EHR)

## Abstract

**Objectives:**

Mood instability is a clinically important phenomenon but has received relatively little research attention. The objective of this study was to assess the impact of mood instability on clinical outcomes in a large sample of people receiving secondary mental healthcare.

**Design:**

Observational study using an anonymised electronic health record case register.

**Setting:**

South London and Maudsley NHS Trust (SLaM), a large provider of inpatient and community mental healthcare in the UK.

**Participants:**

27 704 adults presenting to SLaM between April 2006 and March 2013 with a psychotic, affective or personality disorder.

**Exposure:**

The presence of mood instability within 1 month of presentation, identified using natural language processing (NLP).

**Main outcome measures:**

The number of days spent in hospital, frequency of hospital admission, compulsory hospital admission and prescription of antipsychotics or non-antipsychotic mood stabilisers over a 5-year follow-up period.

**Results:**

Mood instability was documented in 12.1% of people presenting to mental healthcare services. It was most frequently documented in people with bipolar disorder (22.6%), but was common in people with personality disorder (17.8%) and schizophrenia (15.5%). It was associated with a greater number of days spent in hospital (β coefficient 18.5, 95% CI 12.1 to 24.8), greater frequency of hospitalisation (incidence rate ratio 1.95, 1.75 to 2.17), greater likelihood of compulsory admission (OR 2.73, 2.34 to 3.19) and an increased likelihood of prescription of antipsychotics (2.03, 1.75 to 2.35) or non-antipsychotic mood stabilisers (2.07, 1.77 to 2.41).

**Conclusions:**

Mood instability occurs in a wide range of mental disorders and is not limited to affective disorders. It is generally associated with relatively poor clinical outcomes. These findings suggest that clinicians should screen for mood instability across all common mental health disorders. The data also suggest that targeted interventions for mood instability may be useful in patients who do not have a formal affective disorder.

**Strengths and limitations of this study**

- This is the largest study (over 27 000 participants) to investigate the impact of mood instability on clinical outcomes in people with mental illness. The findings demonstrate that mood instability occurs across a wide range of mental disorders, rather than being limited to affective disorders. It is also associated with poorer clinical outcomes, independent of psychiatric diagnosis.
- This is the first study to use an automated information extraction method to acquire data on mood instability from electronic health records. This approach maximises the representativeness of everyday clinical practice and generalisability to people receiving secondary mental healthcare.
- The findings are based on data recorded by clinicians delivering routine mental healthcare who were not specifically seeking to elicit symptoms of mood instability. It is therefore possible that mood instability was not always recognised and documented in electronic health records. If anything, this would lead to an underestimate of its prevalence.
- We collected data on mood instability within 1 month of presentation to mental healthcare services, and did not assess severity or change of mood instability symptoms over time. However, even when restricting analysis to mood instability symptoms experienced within 1 month of presentation, the association with poorer clinical outcomes was evident over a long period of follow-up.

## Introduction

Mood instability is a common presenting symptom for people with a wide variety of mental disorders, with as many as 8 of 10 patients reporting some degree of mood instability during assessment by adult community mental health teams.^1^ Although it has principally been considered as a core feature of borderline personality disorder,^2^ mood instability has also been described in bipolar disorder,^3^ depression^4^ and more recently psychotic disorders.^5^ Across a range of mental disorders, mood instability has been associated with poor functioning, unhappiness and low self-esteem,^6–8^ increased use of healthcare services^9^ and suicidality.^10^

A number of rating scales have been developed to measure mood instability.^11–15^ However, these are not routinely used in clinical practice and the presence of mood instability can be overlooked, particularly as it is sometimes perceived as being limited to affective disorders.^9^ Most research on mood instability has involved samples with a single disorder that may not be representative of the population of patients with mood instability seen in everyday clinical practice.^10^

Clinical information is now widely recorded in the form of electronic health records (EHRs). In the present study, we used a novel information extraction tool to identify the presence of mood instability in a large sample of electronic records collected from individuals with a psychotic, affective or personality disorder.^16^ ^17^ We then examined the relationship between mood instability, mental disorder diagnosis and clinical outcomes. We tested the hypothesis that mood instability is present across a wide range of mental disorders at presentation to mental health services, and is associated with relatively poor clinical outcomes, as indexed by the frequency and duration of mental health inpatient care.

## Methods

### Participants

All individuals aged between 16 and 65 years who presented to the South London and Maudsley NHS Foundation Trust (SLaM) between 1 April 2006 and 31 March 2013 and who received a diagnosis of schizophrenia and related disorders (ICD-10 F2x), bipolar affective disorder (F30 and F31), psychotic depression (F32.3 and F33.3), personality disorder (F60, F61), unipolar depression without psychosis (F32 and F33, excluding F32.3 and F33.3) or any other affective disorder (F34, F38, F39) were included in the study. Applying these inclusion criteria, a sample of 27 704 participants was obtained. Of these, 3221 (11.6%) presented initially to inpatient clinical services. Outcome data were collected up to 31 March 2014. All participants were assessed for outcomes within 1 year of the date of presenting to a mental health service in SLaM. Participants with sufficient follow-up data were also assessed for outcomes within 2 years (presenting between 1 April 2006 and 31 March 2012, n=24 848), 3 years (presenting between 1 April 2006 and 31 March 2011, n=21 188), 4 years (presenting between 1 April 2006 and 31 March 2010, n=17 130) and 5 years (presenting between 1 April 2006 and 31 March 2009, n=13 032).

### Source of clinical data

The study was conducted using the SLaM Biomedical Research Centre (BRC) Case Register.^18^ SLaM is a large provider of mental healthcare in South London, covering a geographic catchment of approximately 1.2 million residents. Since April 2006, SLaM has used a single electronic health record across all clinical services known as the electronic Patient Journey System (ePJS). The SLaM BRC Case Register extracts anonymised clinical data from ePJS including structured fields (for demographic information) and de-identified unstructured free text fields from case notes and correspondence.^18^ A patient-led oversight committee provides governance for all projects conducted using these data.^19^ Healthcare professionals use these free text fields to document clinical information during the course of providing mental healthcare to patients. The clinical information documented includes history, mental state examination, diagnostic formulation and management plan. Data for this study were obtained from these sources of clinical data in the SLaM BRC Case Register using Clinical Record Interactive Search (CRIS), a bespoke database search and assembly tool which has supported a range of studies using this data set.^20–25^

### Mood instability measurement development

The natural language processing (NLP) software package TextHunter^17^ ^26^ was used to extract documentation of mood instability from unstructured free text fields of clinical assessments and correspondence in the SLaM BRC Case Register. On the basis of the rationale that a varied lexicon is used to label and describe symptomatology in healthcare records,^27^ three NLP applications were developed for each of the following affective construct terms: mood, affect and emotion. In order to ascertain the concept of instability, a free text search was conducted on the three keywords (mood, affect and emotion) to identify the most frequently used modifier words up to two words on either side of the keyword. The search results were manually reviewed by TL, RP and MT and modifier words relevant to the concept of instability (including common misspellings) were selected for inclusion in a gazetteer for each of the three NLP applications (see online supplementary table S1). This approach was used in order to develop NLP applications that extracted clinical information relevant to the data on which they were applied.^26^ Although not present in the initial search results, the words ‘instability’, ‘dysfunction’ and ‘irregular’ were also included in all three applications since they are commonly used in the literature to describe mood instability.^15^

All sentences in the SLaM BRC Case Register containing the keywords and modifier words (see online supplementary table S1) were extracted and used as a basis to develop NLP applications to identify the constructs of instability of mood, affect and emotion. For each application, a human annotator (TL) classified the presence or absence of the construct in around 300 sentences to generate a reference data set for subsequent precision testing. The reference data set of each application was also annotated by RP in order to test the inter-annotator agreement for the classification of sentences. Online supplementary table S2 shows the breakdown of annotations and the inter-annotator agreement for each of the three NLP applications. Percentage agreement was above 90% and Cohen's κ at least 0.80 for all applications indicating good inter-annotator agreement in determining each construct. A supervised machine learning approach with active learning was used to identify sentences containing the constructs of interest. Further sentences were classified by a human annotator (TL) to generate a training data set on which a ‘bag-of-words’ support vector machine learning algorithm was applied (with one round of active learning) in order to develop NLP applications to identify each construct.^28^ Each application was tested against the reference data set to obtain baseline precision (positive predictive value) and recall (sensitivity) statistics at a sentence level (see online supplementary figure S1).^29^ As patients with mood instability had multiple sentences in their clinical record which were relevant to the constructs in this study, the NLP applications were developed to maximise the precision of each application in order to reduce the likelihood of false-positive results. A machine learning probability threshold was therefore applied to each application to obtain a per sentence precision (positive predictive value) of at least 90%. This value was determined as the optimum for precision based on previous studies evaluating NLP applications to extract symptom data in mental health.^26^ Online supplementary table S3 shows the precision statistics for each of the three NLP applications. Baseline precision exceeded 80% for all applications. Applying probability thresholds to achieve at least 90% precision resulted in a small reduction in recall for all applications.

Once developed, the applications were then applied to the BRC Case Register and the output of all three were combined to generate a binary variable for each participant defined as any documentation of instability of mood, affect or emotion within 1 month of presentation to SLaM. This variable was used to assess the prevalence of mood instability within the study population and also as the predictor for regression analyses on clinical outcomes described subsequently.

### Clinical outcome measures and covariates

The primary outcome was number of days spent in a psychiatric hospital during the follow-up period. This outcome measure was chosen because the increased duration of hospital stay represents a measure of illness severity as well as a significant impact to individuals, their family and carers and mental healthcare services.^30^ Secondary outcomes included any compulsory hospital admission (under the UK Mental Health Act), frequency of hospital admissions, antipsychotic prescription and non-antipsychotic mood stabiliser prescription during the follow-up period. For the purposes of this study, antipsychotics were defined as any licensed antipsychotic medication listed in section 4.2.1 or 4.2.2 of the British National Formulary (BNF)^31^ and non-antipsychotic mood stabilisers were defined as valproate, carbamazepine, lamotrigine or lithium.^32^ The following variables were extracted as covariates for multivariable analyses: age, gender, ethnicity, marital status and diagnosis. All covariate data obtained were those closest to the date of presenting to SLaM. Ethnicity was recorded according to categories defined by the UK Office for National Statistics.^33^

### Statistical analysis

The data were analysed using Stata (V.12.0).^34^ Descriptive statistics for predictor, covariate and outcome variables were obtained as the mean and variance for number of hospital admissions, mean and SDs for number of days spent in hospital and as frequencies and percentages for all other variables.

The association of mood instability with number of inpatient days was assessed using multiple linear regression. Owing to overdispersion, association of mood instability with number of hospital admissions was analysed using multivariable negative binomial regression. Associations with compulsory hospital admission, antipsychotic prescription and non-antipsychotic mood stabiliser prescription were assessed using multivariable binary logistic regression. Reference groups for covariates in regression analyses were defined as those with the greatest prevalence for each variable. A sensitivity analysis was performed to assess the impact of missing data for marital status which affected 4120 people in the sample.

## Results

### Prevalence and distribution of mood instability

The overall prevalence in our sample of recorded mood instability within 1 month of clinical presentation was 12.1% (table 1). Mood instability was most likely to be present in people who were younger (16–25 years) and female, and less likely in those who were single and who presented with unipolar depression. The strongest diagnostic association of mood instability was seen among those presenting with bipolar disorder. Mood instability was also associated with personality disorder and schizophrenia but to a lesser degree than with bipolar disorder. A sensitivity analysis which only included participants with no missing covariate data (see online supplementary table S4) did not reveal any meaningful differences.

**Table 1:** Binary logistic regression analysis of factors associated with mood instability (n=27 704)

Factor	Group	Number in sample	Prevalence of documented mood instability within 1 month (%)	Association with mood instability			
				Unadjusted		Adjusted model*	
				OR (95% CI)	p Value	OR (95% CI)	p Value
Age (years)	16–25	7133	16.3	1.28 (1.17 to 1.40)	<0.001	1.32 (1.20 to 1.45)	<0.001
	26–35	7842	13.2	Reference		Reference	
	36–45	6611	9.8	0.71 (0.64 to 0.79)	<0.001	0.73 (0.65 to 0.81)	<0.001
	46–55	4066	9.1	0.65 (0.58 to 0.74)	<0.001	0.67 (0.58 to 0.76)	<0.001
	56–65	2052	7.1	0.50 (0.42 to 0.60)	<0.001	0.50 (0.41 to 0.60)	<0.001
Gender	Male	12 532	10.9	0.81 (0.75 to 0.87)	<0.001	0.75 (0.69 to 0.81)	<0.001
	Female	15 172	13.2	Reference		Reference	
Ethnicity	White	15 691	12.5	Reference		Reference	
	Asian	1511	12.6	1.01 (0.86 to 1.18)	0.94	0.93 (0.79 to 1.09)	0.36
	Black	5203	13.3	1.07 (0.98 to 1.18)	0.15	0.95 (0.87 to 1.05)	0.35
	Other	5299	9.8	0.76 (0.69 to 0.84)	<0.001	0.80 (0.72 to 0.89)	<0.001
Marital status (first recorded)	Married/cohabiting	5115	11.7	0.88 (0.80 to 0.97)	0.010	1.16 (1.04 to 1.28)	0.007
	Divorced/separated	2391	11.1	0.82 (0.72 to 0.94)	0.005	1.18 (1.02 to 1.36)	0.028
	Single	16 078	13.1	Reference		Reference	
	Not recorded	4120	9.4	0.69 (0.61 to 0.77)	<0.001	0.82 (0.73 to 0.92)	0.001
Diagnosis	Schizophrenia and related	5860	15.5	2.11 (1.92 to 2.32)	<0.001	2.23 (2.02 to 2.46)	<0.001
	Bipolar affective disorder	2691	22.6	3.37 (3.03 to 3.76)	<0.001	3.42 (3.06 to 3.82)	<0.001
	Psychotic depression	767	14.0	1.87 (1.51 to 2.31)	<0.001	2.00 (1.61 to 2.48)	<0.001
	Personality disorder	2311	17.8	2.50 (2.21 to 2.82)	<0.001	2.39 (2.11 to 2.71)	<0.001
	Unipolar depression (without psychosis)	14 192	8.0	Reference		Reference	
	Other affective disorder	1883	10.6	1.36 (1.16 to 1.60)	<0.001	1.35 (1.15 to 1.59)	<0.001

*Results adjusted for all the factors reported in this table.

### Hospital admission and pharmacological outcomes

Mood instability was associated with a greater number of days spent in hospital, a greater likelihood of compulsory admission to hospital and increased frequency of hospital admission (table 2) up to 5 years following clinical presentation. After adjusting for age, gender, ethnicity, marital status and diagnosis in multivariable regression analyses, mood instability remained a significant predictor of these hospitalisation outcomes (table 3). There was an excess of zero values for the number of hospital admissions during the follow-up period. However, despite a significant Vuong test result, fitting a zero-inflated negative binomial regression model (see online supplementary table S5) resulted in only a slight reduction in incident rate ratios compared with standard negative binomial regression (table 3). Mood instability was also associated with an increased risk of antipsychotic prescription and non-antipsychotic mood stabiliser prescription (table 4). Much of the increased risk of antipsychotic prescription occurred within the first year of follow-up while the cumulative risk of non-antipsychotic mood stabiliser prescription increased steadily over the period of 5-year follow-up. These associations remained after adjusting for demographic factors in multivariable logistic regression analyses (table 5).

**Table 2:** Hospital admission outcomes among individuals with and without documented mood instability

		Mean number of inpatient days (SD)		Compulsory admission (%)		Mean number of admissions (variance)	
Follow-up period (months)	Number in sample	History of mood instability	No history of mood instability	History of mood instability	No history of mood instability	History of mood instability	No history of mood instability
0–12	27 704	25.1 (50.7)	8.6 (35.9)	28.5	7.4	0.63 (0.71)	0.21 (0.28)
0–24	24 848	32.7 (77.3)	13.9 (60.3)	29.3	9.0	0.72 (1.10)	0.26 (0.47)
0–36	21 188	38.6 (97.5)	18.0 (79.4)	30.0	9.9	0.82 (1.48)	0.31 (0.67)
0–48	17 130	45.5 (119.5)	21.7 (92.4)	30.1	10.9	0.90 (2.02)	0.37 (0.93)
0–60	13 032	53.1 (138.6)	25.5 (104.9)	30.5	12.0	0.98 (2.44)	0.43 (1.19)

**Table 3:** Multivariable analyses of relationship between mood instability and frequency of hospital admission, likelihood of compulsory hospital admission and mean number of days spent in hospital up to 5 years following presentation to mental health services

Follow-up period (months)	Number in sample	Number of days spent in hospital* β Coefficient (95% CI), p value	Compulsory hospital admission† OR (95% CI), p value	Number of admissions to hospital‡ Incidence rate ratio (95% CI), p value
0–12	27 704	13.4 (12.1 to 14.8), <0.001	4.55 (4.11 to 5.04), <0.001	2.62 (2.47 to 2.77), <0.001
0–24	24 848	13.9 (11.4 to 16.3), <0.001	3.77 (3.39 to 4.20), <0.001	2.33 (2.18 to 2.49), <0.001
0–36	21 188	13.5 (10.0 to 17.1), <0.001	3.39 (3.01 to 3.81), <0.001	2.17 (2.01 to 2.35), <0.001
0–48	17 130	15.9 (11.2 to 20.7), <0.001	3.02 (2.64 to 3.45), <0.001	2.07 (1.89 to 2.26), <0.001
0–60	13 032	18.5 (12.1 to 24.8), <0.001	2.73 (2.34 to 3.19), <0.001	1.95 (1.75 to 2.17), <0.001

Results adjusted for age, gender, ethnicity, marital status and diagnosis.

*Multiple linear regression.

†Multivariable logistic regression.

‡Multivariable negative binomial regression.

**Table 4:** Cumulative percentage of patients with and without documented mood instability who were subsequently prescribed an antipsychotic or non-antipsychotic mood stabiliser

		Antipsychotic prescription (%)		Non-antipsychotic mood stabiliser prescription (%)	
Follow-up period (months)	Number in sample	History of mood instability	No history of mood instability	History of mood instability	No history of mood instability
0–12	27 704	52.5	27.8	19.8	8.0
0–24	24 848	53.7	30.7	22.0	9.5
0–36	21 188	54.8	32.5	24.0	10.6
0–48	17 130	55.7	34.3	25.1	12.0
0–60	13 032	56.1	35.8	27.6	12.7

**Table 5:** Multivariable logistic regression analyses of relationship between mood instability and likelihood of antipsychotic and non-antipsychotic mood stabiliser prescription up to 5 years following presentation to mental health services

Follow-up period (months)	Number in sample	Antipsychotic prescription OR (95% CI), p value	Non-antipsychotic mood stabiliser prescription OR (95% CI), p value
0–12	27 704	2.71 (2.48 to 2.96), <0.001	2.26 (2.03 to 2.52), <0.001
0–24	24 848	2.40 (2.18 to 2.64), <0.001	2.09 (1.86 to 2.33), <0.001
0–36	21 188	2.24 (2.01 to 2.50), <0.001	2.06 (1.82 to 2.32), <0.001
0–48	17 130	2.14 (1.89 to 2.43), <0.001	1.90 (1.66 to 2.17), <0.001
0–60	13 032	2.03 (1.75 to 2.35), <0.001	2.07 (1.77 to 2.41), <0.001

Antipsychotic: any licensed antipsychotic medication listed in section 4.2.1 of the British National Formulary (BNF).

Non-antipsychotic mood stabiliser: valproate, carbamazepine, lamotrigine or lithium.

Results adjusted for age, gender, ethnicity, marital status and diagnosis.

## Discussion

To the best of our knowledge, this is the first study to investigate mood instability as documented in the health records of people with mental illness. We demonstrate that it is possible to identify the presence of mood instability in electronic health records using automated NLP methods. Using a data-driven approach which was tailored to the clinical records in the SLaM BRC Case Register, we developed applications with a high degree of accuracy and inter-rater reliability. As a result, we were able to implement rapid extraction of data on mood instability from a very large sample of patients (27 704 in our study) that would have been logistically unfeasible by either a manual review of clinical records or through *prospective* data collection.

As hypothesised, we found that mood instability is frequently documented in people across a range of different mental disorders (12.1% in our sample). Although this is comparable to the overall prevalence found in other studies (13.2% in Black *et al*;^35^ 13.9% in Marwaha *et al*^9^), these were measured in general populations, whereas our participants were defined by their use of mental health services. Prevalences of mood instability between 49.2% and 83.8% have been reported in other studies,^1^ ^5^ ^9^ ^10^ but these findings were based on patient self-report measures: in this study, mood instability was measured by its written presence in clinical records. As specific rating scales to measure mood instability are not routinely applied in clinical practice, the lower prevalence seen in our study could indicate that symptoms of mood instability are not always elicited or documented in electronic health records, and when they are documented because they are deemed to be clinically relevant to the patient's care. However, it is possible that if clinicians had specifically sought to identify the presence of mood instability using screening questionnaires, the prevalence may have been higher than that elicited using NLP on routinely recorded clinical data. Furthermore, the documentation of symptoms may have been biased by the underlying diagnosis. This could be investigated further in future studies comparing NLP methods with standardised questionnaires for eliciting mood instability and mental disorder diagnosis.

Patients with documented mood instability were more likely to be young, female and single, largely consistent with findings from a previous study investigating the prevalence of mood instability in a large adult population.^9^ Mood instability was particularly associated with a diagnosis of bipolar affective disorder. This finding corroborates previous research which has indicated that mood instability is a key factor in bipolar disorder, as distinct from episodes of mania and depression.^36^ ^37^ However, mood instability was also prevalent in other disorders (such as schizophrenia, psychotic depression and personality disorders), suggesting that it occurs in a range of mental disorders, consistent with recent findings from British National Survey data.^5^

The data supported the hypothesis that mood instability is associated with poorer clinical outcomes and increased use of healthcare services. Those with a recorded instance of mood instability within 1 month of presentation to mental health services were admitted to hospital more frequently and were at greater risk of being compulsorily detained under the UK Mental Health Act over the 5-year follow-up period. Furthermore, people with mood instability were likely to spend a significantly greater time in hospital (around 13 additional days within the first year following presentation). The increased risk of hospitalisation outcomes was greatest in the first year following presentation, indicating the significant impact of mood instability on initial clinical outcomes after presenting to mental health services, independent of psychiatric diagnosis. Extensive use of inpatient resources has been well observed in patients with mood instability,^9^ and this represents morbidity to individuals and cost to healthcare services.^38^ Consequently, direct treatment of this symptom, irrespective of a patient's working diagnosis, could have considerable health economic benefits.

The presence of mood instability was also associated with an increased likelihood of antipsychotic and non-antipsychotic mood stabiliser prescription. Our data suggest that the greatest rate of antipsychotic prescribing occurred within 1 year of follow-up while the cumulative risk of non-antipsychotic mood stabiliser prescriptions progressively increased over 5 years of follow-up. Multivariable regression analysis demonstrated that these associations were also independent of psychiatric diagnosis. This suggests that mood instability was associated with early antipsychotic treatment, consistent with their utility as rapid and effective mood stabilisers,^39^ ^40^ followed by the subsequent use of lithium or anticonvulsants to provide longer term mood stabilisation. However, as our findings were drawn from observational data, it is not possible to infer an aetiological association between mood instability and pharmacotherapy. It is possible that this finding represents the choice of pharmacotherapy in relation to the licensed indication for the underlying disorder being treated rather than specifically to treat symptoms of mood instability.

A major strength of the study was the substantial size of the sample. Participants were gathered from the case register of a large mental healthcare provider and included based on contact with services within a given period, rather than being specially selected for research purposes. This approach maximised the generalisability of our findings since the sample was more representative of everyday clinical practice. Another strength was the use of a novel automated information extraction method to reliably and accurately ascertain the presence of documented mood instability, thereby reducing any potential bias which may occur through a manual review of case records by multiple investigators.

There were some limitations to this study which could be addressed in future research. As the data were drawn from routine clinical records, it was found that some participants had missing data for marital status. However, a sensitivity analysis including only participants with full covariate data did not reveal any meaningful differences in results. There were also other covariates of interest which were not comprehensively documented in electronic health records (and consequently could not be analysed) including the presence and severity of manic and psychotic symptoms, history of deliberate self-harm, age of onset of illness and drug and alcohol misuse.

A further limitation of using routine clinical records was the impact of loss to follow-up. Whereas in a prospective observational or interventional study there is a standardised schedule to obtain follow-up data from participants, this is not the case for data from routine clinical care where contact with mental health services is determined by a complex interaction of patient and service related factors. It is possible that patients were discharged from mental health services during the period of the study for a number of reasons including improvement in symptoms (ie, planned discharge to primary care), disengagement from mental health services and moving outside the catchment area of SLaM. It was not possible to obtain data on the reason for discharge in our data set to see if there was an association with mood instability which could have biased outcomes. Further work is needed to establish the impact of mood instability on level of engagement with mental health services.

It was decided to limit observations of mood instability to within 1 month of contact with services. It may be that patients develop or display this problem further into their treatment, meaning that some instances of mood instability may have been overlooked. However, it was noteworthy that even restricting the ascertainment of mood instability to this time window resulted in substantial associations with poorer clinical outcomes over the period of follow-up of up to 5 years. Also, in order to balance project scope and feasibility, the sample was limited to patients with psychotic and affective disorders which have been shown to be relevant to mood instability in previous studies.^1^ ^2^ ^4^ ^5^ However, mood instability is also known to occur in some disorders not included in this study (eg, attention deficit hyperactivity disorder).^41^ Future work could expand on other diagnostic categories to assess the impact of mood instability in other mental disorders.

The definition and measurement of mood instability in our study conceptualised the construct as a binary variable (present or absent) and did not collect data on the frequency or severity of the instability, which may be important to predict future illness course.^42^ ^43^ It also combined data from three separate applications which focused on instability related to distinct affective terms (mood, affect and emotion). This method was chosen based on findings from previous studies which indicate that these three terms may be used interchangeably despite representing subtly different constructs.^5^ ^9^ ^15^ This approach raises questions about the construct validity of the mood instability measure since it is not certain that the examples identified by each tool are clinically or phenomenologically equivalent. Nonetheless, analysis of the large quantity of data obtained using this study's measure of mood instability led to meaningful and clinically relevant findings, indicating that it is a robust research tool which targets an important construct in its own right, despite its potential heterogeneity.

## Conclusion

Taken together, our findings suggest that mood instability is associated with poorer clinical outcomes and increased use of antipsychotic and non-antipsychotic mood stabiliser therapy, regardless of the mental disorder with which an individual initially presents. Our study suggests that clinicians should consider screening for the presence of mood instability on a routine basis and that it should be given more attention, irrespective of an individual's underlying psychiatric diagnosis. These findings have important implications for clinical practice and highlight the need for interventional studies across a range of mental disorders to better understand which pharmacological and psychosocial interventions are most successful in reducing the impact of mood instability.
